# Supplementary material for: Explosive eruption style modulates volcanic electrification signals
Source: Commun Earth Environ. 2024 Jul 9;5(1):367. doi: 10.1038/s43247-024-01520-6 (PMC11233269; doi:10.1038/s43247-024-01520-6)
Supplement: Supplementary file 2 — Supplementary Information [file 43247_2024_1520_MOESM2_ESM.pdf]

Supplementary Information

Explosive eruption style modulates volcanic electrification signals

Caron E. J. Vossen[1,*], Corrado Cimarelli[1], Luca D'Auria[2,3], Valeria Cigala[1], Ulrich Kueppers[1], José Barrancos[2,3], Alec J. Bennett[4,5]

[1] Department of Earth and Environmental Sciences, Ludwig-Maximilians-Universität München, Munich, Germany

[2] Instituto Volcanológico de Canarias (INVOLCAN), 38320 San Cristóbal de La Laguna, Tenerife, Canary Islands, Spain

[3] Instituto Tecnológico y de Energías Renovables (ITER). Polígono Industrial de Granadilla, s/n 38600 - Granadilla de Abona, Santa Cruz de Tenerife, Spain

[4] Bristol Industrial and Research Associates Ltd (Biral), Unit 8 Harbour Road Trading Estate, Portishead, Bristol, BS20 7BL, United Kingdom

[5] Department of Electronic and Electrical Engineering, University of Bath, Bath, United Kingdom

[*] Corresponding author. E-mail address: caron.vossen@min.uni-muenchen.de

Supplementary Methods

In this study, the volcanic tremor is considered within two frequency bands: Very-Long-Period (VLP 0.4-0.6 Hz) and Long-Period (LP 1-5 Hz). Using the Principal Component Analysis (PCA) on the log-normalised tremor amplitude values, the temporal variation of these two components is analysed. Supplementary Figure 1 shows the result of the PCA data decomposition. The first principal component (PC1) is mostly related to the absolute amplitude, while the second principal component (PC2) mostly depends on their ratio.

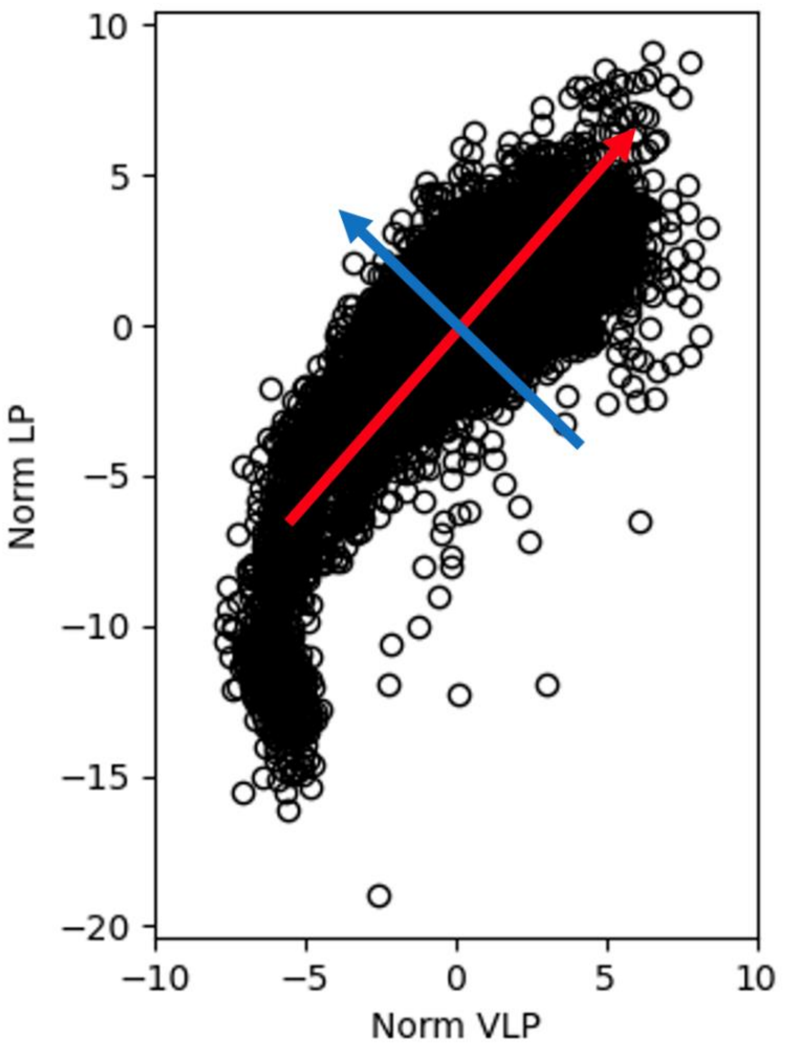

Supplementary Figure 1: ***Graphical representation of the PCA data decomposition.*** *The whole dataset is represented with black circles as a function of the log-normalized VLP and LP amplitudes. The eigenvectors representative of the two PCA components are shown as red (PC1, first principal component) and blue (PC2, second principal component) arrows.*

Temperature data from weather balloons released at Güímar (station 60018) on Tenerife, Canary Islands (Spain), is provided by the University of Wyoming, Department of Atmospheric Science (http://weather.uwyo.edu/), which are used to construct the 0°C, -10°C and -20°C isotherms (Figure 1 in main text). To ascertain, however, that these measurements represent the conditions on La Palma as well, the data was compared to temperature measurements from two fixed weather stations on La Palma, at El Paso (altitude of 844 m) and Roque de los Muchachos (altitude of 2223 m) carried out by the State Meteorological Agency (AEMET) of Spain. The comparisons are shown in Supplementary Figures 2 and 3 for El Paso and Roque de los Muchachos, respectively. Although the temperature difference between night and day is greater for the local stations on La Palma (AEMET), the overall trend is very similar between the two data sets. Therefore, we concluded that the Güímar data is sufficiently accurate to construct the different isotherms.

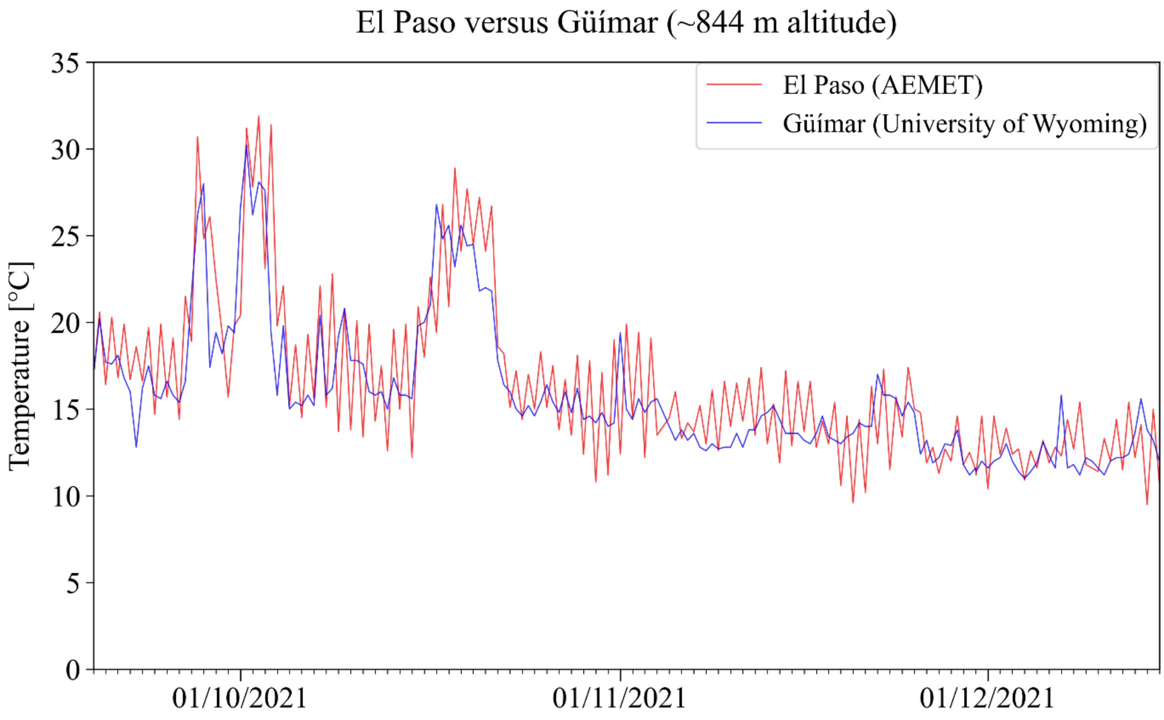

Supplementary Figure 2: ***Comparison of temperature measurements at El Paso, La Palma, and Güímar, Tenerife, at ~844 m altitude.*** *The temperature at El Paso (red line) is measured each day at 00:00 and 12:00 UTC by a fixed weather station operated by the State Meteorological Agency (AEMET) of Spain. The temperature data of Güímar (station 60018, blue line) is obtained from weather balloons that are released twice a day (at 00:00 and 12:00 UTC) and is provided by the University of Wyoming.*

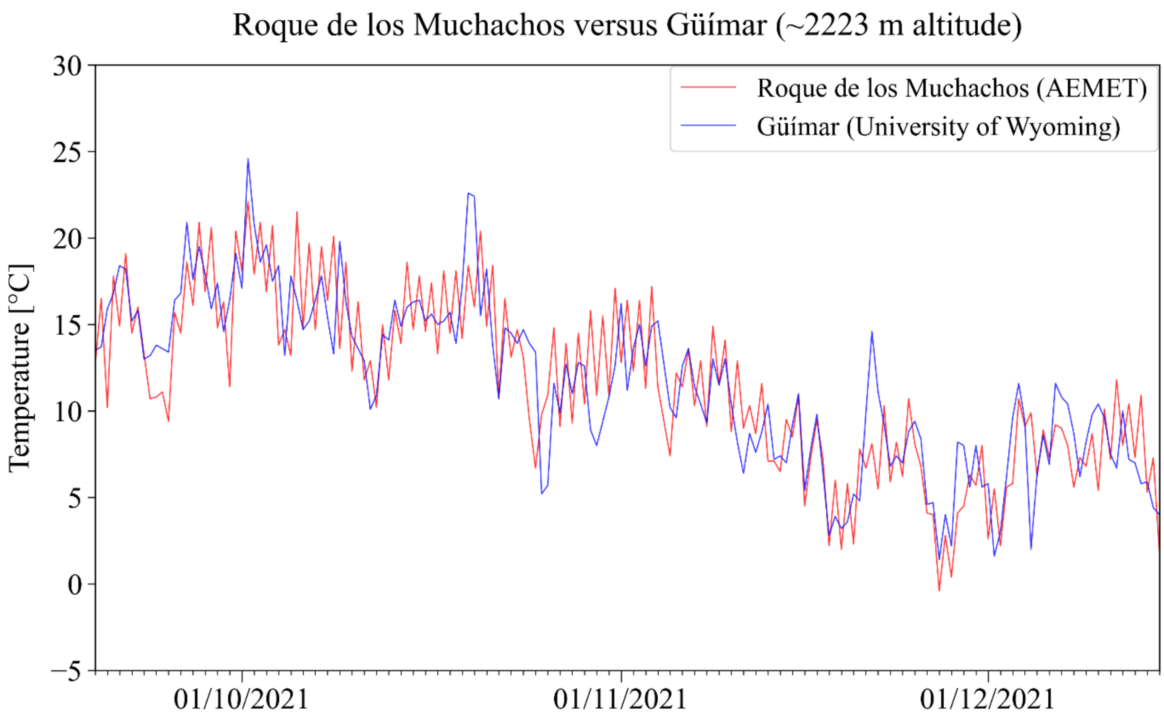

Supplementary Figure 3: ***Comparison of temperature measurements at Roque de los Muchachos, La Palma, and Güímar, Tenerife, at ~2223 m altitude.*** *The temperature at Roque de los Muchachos (red line) is measured each day at 00:00 and 12:00 UTC by a fixed weather station operated by the State Meteorological Agency (AEMET) of Spain. The temperature data of Güímar (station 60018, blue line) is obtained from weather balloons that are released twice a day (at 00:00 and 12:00 UTC) and is provided by the University of Wyoming.*

Supplementary Results

The electrical activity recorded on 3 and 4 November 2021 is provided in Supplementary Figure 4 together with close-up plots of the different electrical signatures that were detected chronologically. Supplementary Figure 5 displays the difference between two phases of lava fountaining observed during the study case of 3-4 November. Lava fountaining on the evening of 3 November generated a relatively dense, turbulent 3-km tall ash plume. The initial diameter of the eruption column varied between 300-500 m. In comparison, a lava fountaining phase on the evening of 4 November emitted less ash and did therefore not form such a dense ash plume. Instead, the initial diameter of the eruption column was smaller (100-200 m).

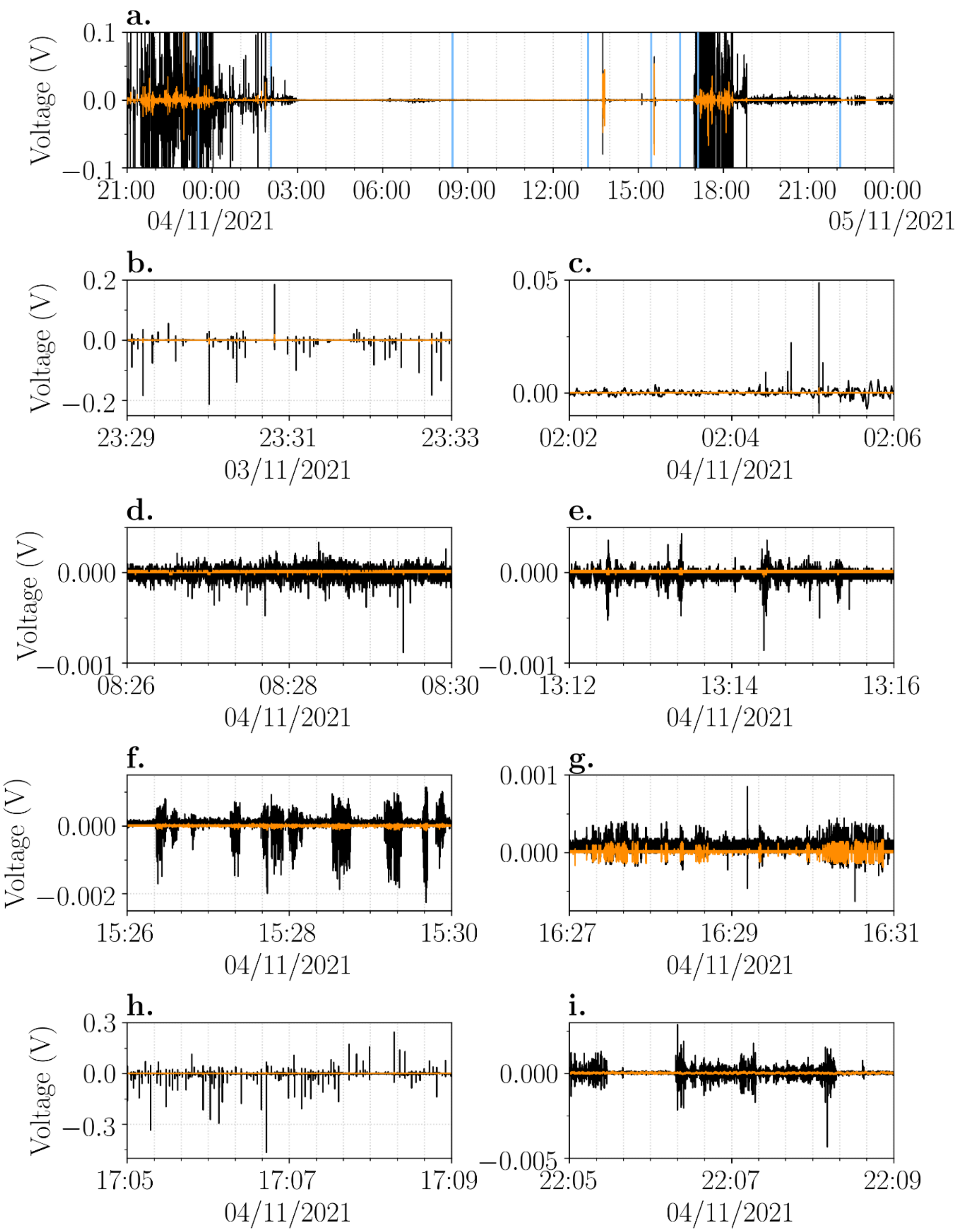

Supplementary Figure 4: ***Full signal and snapshots of dominant electrical activity observed in chronological order on 3 and 4 November 2021.*** *All panels show the voltage (V) measured by the primary (black line) and secondary (orange line) antennas of BTD2.* ***a)*** *Electrical activity measured between 21:00 UTC on 3 November and 00:00 UTC on 5 November 2021. This panel is the same as Figure 4b in the main text, but with a smaller y-axis to visualise the*

difference in amplitude of the various electrical signals better. The blue vertical bars indicate the time windows of the snapshots shown in panels ***b-i****.* ***b)*** *Individual high-amplitude electrical discharges (type 1 signal);* ***c)*** *Less frequent electrical discharges (type 1 signal) with measured voltages an order of magnitude lower than in panel* ***b****. Due to the lower magnitude of the electrical discharges, the movement of charge becomes visible in the electrical data (type 5 signal);* ***d)*** *Faint electrical discharges that remained mostly undetected by the volcanic lightning detection algorithm (type 4 signal);* ***e)*** *Bursts of quasi-continuous electrical discharges lasting ~3-10 seconds long (type 3 signal);* ***f)*** *Burst of quasi-continuous electrical discharges of slightly longer duration (up to ~45 seconds) than in panel* ***e*** *(type 3 signal);* ***g)*** *Ash fall at the sensor, producing electrical signals of opposite polarity at the two antennas (type 6 signal);* ***h)*** *Individual high-amplitude electrical discharges (type 1 signal);* ***i)*** *Minutes-long burst of quasi-continuous electrical discharges (type 2 signal). Note the different scale on the y-axis for each panel.*

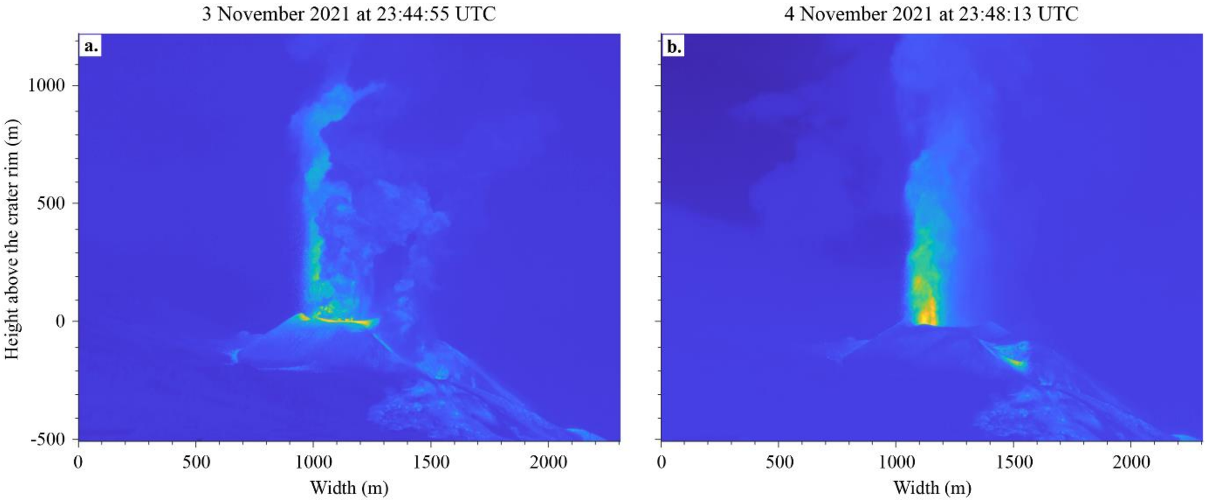

Supplementary Figure 5: ***Thermal infrared frames showing two different phases of lava fountaining.*** ***a)*** *Ash-rich lava fountaining at the main vent producing relatively dense, turbulent eddies and a 3-km tall ash plume at 23:44:55 UTC on 3 November 2021. A second vent is currently active on the lower right.* ***b)*** *Lava fountaining at 23:48:13 UTC on 4 November 2021, emitting less ash in comparison to the explosive activity on the evening of 3 November shown in panel* ***a****. As a result, no dense ash plume was formed.*
